# Feasibility of US-CT image fusion to identify the sources of abnormal vascularization in posterior sacroiliac joints of ankylosing spondylitis patients

**DOI:** 10.1038/srep18356

**Published:** 2015-12-16

**Authors:** Zhenlong Hu, Jiaan Zhu, Fang Liu, Niansong Wang, Qin Xue

**Affiliations:** 1 Department of Ultrasound, Shanghai Jiao Tong University Affiliated Sixth People’s Hospital, No. 600 Yishan Road, Shanghai, 200233, People’s Republic of China; 2 Shanghai Institute of Ultrasound in Medicine, No. 600 Yishan Road, Shanghai, 200233, People’s Republic of China; 3 Department of Ultrasound, Peking University People’s Hospital, No. 11 Xizhimen South Street, Beijing, 100044, People’s Republic of China; 4 Department of Rheumatology, Shanghai Jiao Tong University Affiliated Sixth People’s Hospital, No. 600 Yishan Road, Shanghai, 200233, People’s Republic of China

## Abstract

Ultrasound (US) can be used to evaluate the inflammatory activity of the sacroiliac joints (SIJs) in ankylosing spondylitis (AS) patients, but to precisely locate the abnormal vascularization observed on color Doppler US (CDUS) was difficult. To address this issue, we performed US and computed tomography (CT) fusion imaging of SIJs with 84 inpatients and 30 controls, and then assessed the sources of abnormal vascularization in the posterior SIJs of AS patients based on the fused images. Several possible factors impacting the fusion process were considered including the lesion classes of SIJ, the skinfold thickness of the sacral region and the cross-sectional levels of the first, second and third posterior sacral foramina. Our data showed high image fusion success rates at the 3 levels in the AS group (97.0%, 87.5% and 79.8%, respectively) and the control group (96.7%, 86.7%, and 86.7%, respectively).The skinfold thickness was identified as the main factor affecting the success rates. The successfully fused images revealed significant differences in the distribution of abnormal vascularization between 3 levels, as detected via CDUS (P = 0.011), which suggested that inflammation occurred in distinct tissues at different levels of the SIJ (intraligamentous inflammation in Regions 1 and 2; intracapsular inflammation in Region 3).

Within the past decade, ultrasound (US), particularly color Doppler US (CDUS), has become an established imaging technique for rheumatic diseases that is currently indispensable clinically^1^,^2^,^3^,^4^. For ankylosing spondylitis (AS), a chronic inflammatory rheumatic disease that represents the most common spondyloarthropathy, CDUS displays definite value for assessing the inflammatory activity of the sacroiliac joint (SIJ)^5^,^6^,^7^,^8^. This application was first reported in 1999 by Halil Arslan^8^, who indicated that CDUS can reveal increased vascularization and a decreased vascular resistance index (RI) in the posterior regions of the SIJs in AS patients with active sacroiliitis. Subsequent studies, including our previous work, have supported the results of Halil Arslan and have demonstrated that CDUS can be used for the diagnosis and the follow-up of AS patients^5^,^9^. However, evaluating the positions and the sources of abnormal vascularization (blood flow signals with a low RI) solely via US is difficult because the SIJ is positioned obliquely and has an auricular shape; thus, the US beam cannot easily pass through the SIJ to obtain an image of the entire joint. Therefore, new techniques are needed to address this problem.

Fusion imaging is a new technology that creates new images using data from different modalities with geometric consistency. In fusion US (FUS), real-time US images are simultaneously presented with corresponding images obtained from previously acquired CT or magnetic resonance (MR) images^10^. Reports have indicated that image fusion is valuable in cases of US-guided targeted ablation of liver and prostate tumors^11^,^12^,^13^. However, applications of image fusion to examine the musculoskeletal system have rarely been reported.

In this study, we aimed to verify the feasibility of fusion imaging of the SIJs of AS patients and used this technology to locate the active inflammatory activity as visualized by CDUS in these patients to confirm the sources of abnormal vascularization. In this preliminary study, we used CT as the second modality.

## Results

### Subjects

The clinical characteristics of the patients and the controls are summarized in Table 1. High image fusion success rates were observed at all 3 SIJ levels in the AS group (97.0%, 87.5% and 79.8%) and the control group (96.7%, 86.7%, and 86.7%). However, no significant differences were observed between the two groups at the first, second or third SIJ level (P = 0.891, P = 0.886, and P = 0.236, respectively). Furthermore, no significant differences in the skinfold thickness of the sacral region at any level were observed between the two groups. In the AS group, the means and standard deviations of the skinfold thickness of the sacral region among successful cases were 8.76 ± 2.48 mm, 9.05 ± 2.24 mm and 9.28 ± 2.11 mm at the first, second and third SIJ levels, respectively (Table 2). These values were significantly different from those of the failed cases (all P<0.001). Furthermore, a difference in the mean skinfold thickness at each of the 3 SIJ levels was observed between the successful and failed cases in the control group. As shown in Table 3, in the AS group, the differences in the imaging success rates between the 4 classes of SIJ lesions were not significant at any level (P = 0.136, P = 0.708, and P = 0.311 for levels 1, 2, and 3, respectively).

Among the successfully fused US-CT images of the SIJs in the AS group (level 1: 163, level 2: 147, and level 3: 134), CDUS revealed abnormal blood flow signals in 128 SIJs surrounding level 1 (Region 1), 105 surrounding level 2 (Region 2), and 61 surrounding level 3 (Region 3). The distributions of the abnormal blood flow signals surrounding and within the SIJs are presented in Table 4. These data indicated that abnormal blood flow signals were significantly more likely to occur surrounding the SIJs than within Region 1 or 2; conversely, abnormal signals were more likely to occur within Region 3 than surrounding the SIJs. The differences in abnormal blood flow signals among the 3 regions were significant (χ^2^ = 9.101, P = 0.011).

### Cadavers

**Figure 1:**
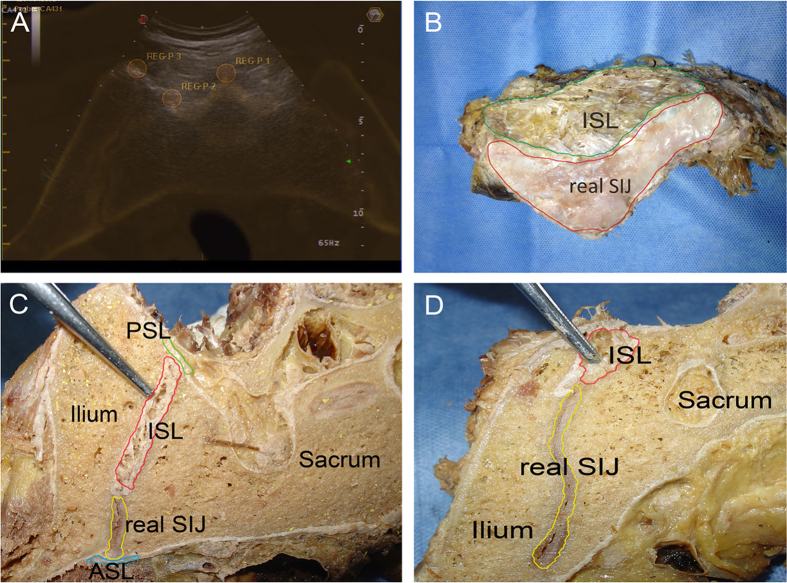
(**A**) A fused image of the SIJ: REG-P1 represents the median sacral crest; REG-P2 represents the left intermediate sacral crest; and REG-P3 represents the bony dorsal border of the left iliac bone. (**B**) An opened SIJ reveals the distribution of the actual SIJ and the ISL. The ISL is the main component of the posterosuperior portion of the SIJ (green highlighted area), whereas the actual synovial joint of the SIJ is located anteriorly (red highlighted area). (**C**) A cross-section of the SIJ at a higher level reveals the ligaments of the SIJ and the actual joint: the anterior sacroiliac ligament (ASL, blue highlighted area), the posterior sacroiliac ligament (PSL, green highlighted area), and the interosseous sacroiliac ligament (ISL, red highlighted area). At a higher level, the ISL is the main component of the SIJ, and the actual synovial joint of the SIJ is located anteriorly and represents a small proportion of the tissue (yellow highlighted area). (**D**) A cross-section of the SIJ at a lower level. Compared with Fig. 2B, the actual joint of the SIJ (red highlighted area) gradually increases in size from the higher level to the lower level. Conversely, the ISL (yellow highlighted area) decreases in size from the higher level to the lower level.

**Figure 2:**
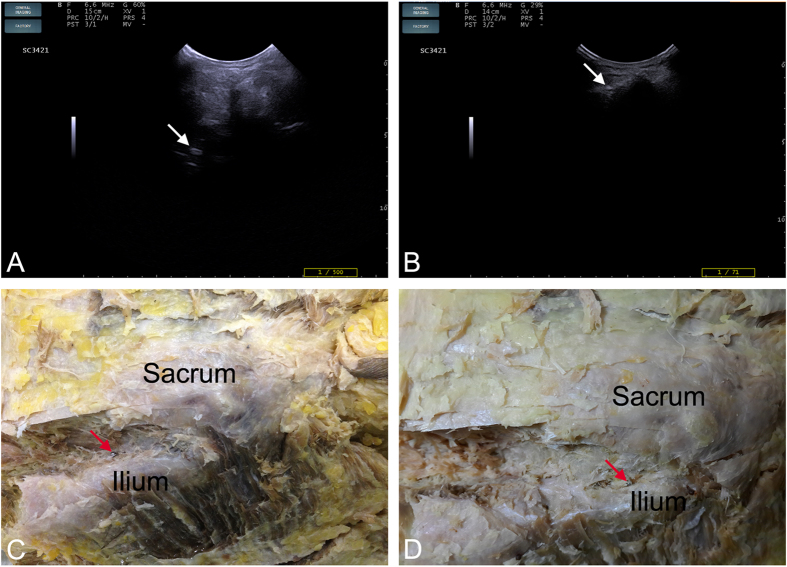
Interventional and dissection tests showing the positions of steel balls at different levels of the SIJ. **(A)** A steel ball visible by US in the higher level of the SIJ (white arrow). **(B)** A steel ball visible by US in the lower level of the SIJ (white arrow). (**C**) The steel ball shown in Fig. 2A was located in the ligamentous portion of the SIJ (red arrow). **(D)** The steel ball shown in Fig. 2B was located in the synovial membrane portion of the SIJ (red arrow).

**Figure 3:**
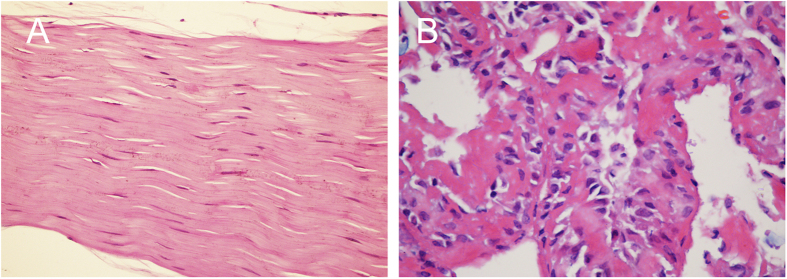
Sections of tissues surrounding the steel ball (HE staining, magnification 400×). (**A**) The tissues surrounding the steel ball shown in Fig. 2C are ligaments. (**B**) The area surrounding the steel ball shown in Fig. 2D consists of synovialis.

The anatomy of two pelvises was examined. One SIJ was opened to expose the full view of the SIJ, and this procedure revealed that the actual synovial joint of the SIJ was positioned anteriorly and that the interosseous sacroiliac ligament (ISL) was the main connection at the posterosuperior portion of the SIJ (Fig. 1B). The other SIJ was cut into cross-sectional slices, and this procedure revealed that the actual synovial joint of the SIJ gradually increased in size from its superior region to its inferior region but that the ligaments decreased in size at the back of the SIJ (Fig. 1B–D). An interventional test and a histological examination were performed on the third SIJ; US indicated that regardless of whether steel balls were placed superficially or deep, the tissues surrounding the balls at levels 1 and 2 were ligaments, whereas the tissue at level 3 was synovial membrane (Figs 2 and 3).

## Discussion

In this study, we demonstrated that image fusion of US and CT can be used to locate abnormal blood flow signals as visualized by CDUS in the SIJs of AS patients with active sacroiliitis, which help to confirm the sources of abnormal vascularization. These abnormal blood flow signals at different levels of the SIJ indicate that inflammation occurred in different tissues of the posterior SIJ. These findings are important because they improve our understanding of imaging performance to examine active sacroiliitis and because this technique may aid in the diagnosis of AS patients, particularly at the early stages of AS.

A successfully fused image of the SIJ (US and CT) is the precondition for locating the abnormal vascularization visualized by CDUS. Klauser *et al.* first reported the use of US-CT image fusion to enhance the accuracy of intra-articular injections into SIJs in 10 cadavers and 10 patients^14^. That study reported a 100% success rate of imaging fusion in the SIJs. Our study provided a larger sample size and a detailed method to assess the imaging success rates. The results revealed that high success ratios were obtained in both the AS and control groups, similar to Klauser’s study, but that no significant differences were observed between AS patients and controls at level 1, 2, or 3. Moreover, in the AS group, the difference in the imaging success rate between the 4 classes of SIJ lesions was not significant. This may be because the SIJ lesions in AS patients predominantly occur within the joints; therefore, registration points located on the surface of the SIJ are less severely affected. In both the AS and control groups, the mean skinfold thicknesses of the sacral region in successful cases were significantly different from those in failed cases at all 3 levels. This difference likely occurred because the thinner skinfold thickness decreased the effective contact area of the convex array US probe, which affected the view of the SIJ via US and thus impacted the registration procedure and caused image fusion failure. These results indicate that the skinfold thickness of the sacral region may be a crucial factor affecting the successfulness of image fusion for the visualization of the SIJ.

Fully understanding the anatomy of the SIJ is important for investigating the location of abnormal vascularization in the SIJ. The SIJ is an auricular-shaped synovial joint^15^. In fact, only the anterior third of the interface between the sacrum and the ilium is a true synovial joint; the remaining junction is composed of extensive ligamentous connections^16^,^17^, including the anterior sacroiliac ligament (ASL), the posterior sacroiliac ligament (PSL), and the ISL. Our analysis of two pelvises revealed that the ISL is the main connection at the posterosuperior portion of the SIJ. More importantly, in cross-sections, the actual synovial joint of the SIJ gradually increased from the superior region to the inferior region. Conversely, the ISL decreased from the superior region to the inferior region (Fig. 1C,D). The interventional test and the histological examination further confirmed the differences in soft tissue between different levels of the posterior portion of the SIJ (Figs 2 and 3). These results are of utmost importance when using CDUS to locate the positions of abnormal blood flow signals and to determine their sources.

Previous studies have indicated that CDUS is valuable for diagnosing active sacroiliitis because this modality can reveal increased vascularization near the posterior portion of the SIJ, as this vascularization presents as blood flow signals with a low RI^5^,^6^,^7^,^8^. Arslan’s study in 1999 suggested that abnormal blood flow signals on CDUS were more frequently observed surrounding the SIJ than within the SIJ^8^. However, we could not identify any additional studies that investigated the exact positions and sources of these signals. Our findings demonstrate that at superior levels of the SIJ (Regions 1 and 2), the vast majority of abnormal blood flow signals are located surrounding the SIJ. This result supports the findings of Arslan. However, at inferior levels of the SIJ (Region 3), the signals were predominantly detected within the SIJ (P < 0.05).

**Figure 4:**
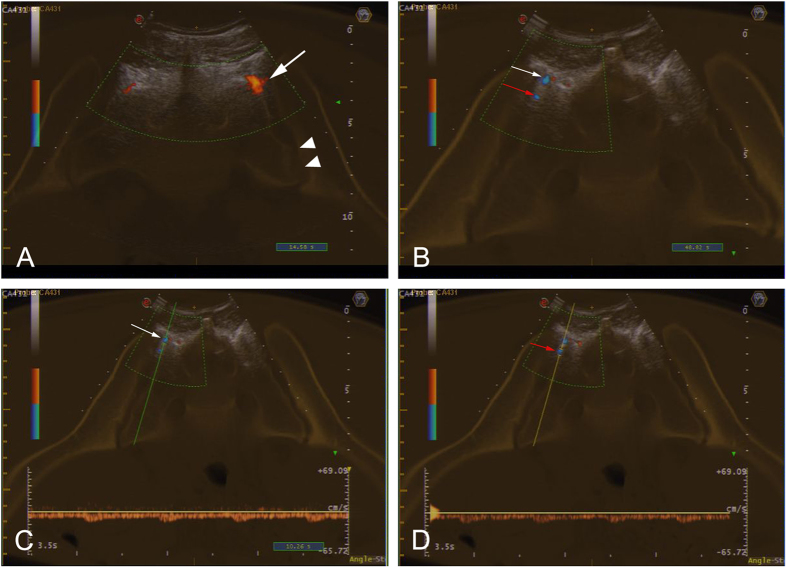
The positions of abnormal blood flow signals. (**A**) Abnormal blood flow signals were located in the ligamentous portion of the SIJ in the cleft (surrounding the SIJ) between the sacrum and the ilium at a higher level (white arrow); the actual synovial joint was in a deeper location (arrow head). (**B**) Abnormal signals within and surrounding the SIJ at a lower level (red arrow and white arrow, respectively) were located at the synovialis and the ligamentous portions of the SIJ, respectively. **(C)** The signal surrounding the SIJ reflects a reduction in resistance on CDUS, perhaps due to enthesitis of the ISL or the PSL. **(D)** The signal within the SIJ reflects a reduction in resistance on CDUS, which may indicate synovitis of the SIJ.

Combined with our anatomical studies, the fused images clearly revealed the positions of the abnormal blood flow signals. Figure 4A presents a blood flow signal with a decreased RI located in the cleft (surrounding the SIJ) between the sacrum and the ilium in Region 1; the tissue in the cleft is the ISL (black arrow). Because of the actual synovial joint is located much deeper at this level (arrow head), US cannot provide a useful view of the synovialis at this level of the SIJ. Thus, the abnormal signal detected in this image is clearly located at the ISL. Our results indicated that nearly all abnormal signals within and surrounding the SIJs at the higher levels (Regions 1 and 2) were located in the ligamentous regions of the SIJs and that these signals were most likely caused by enthesitis of the ISL or the PSL. However, in Region 3, the cleft between the sacrum and the ilium is barely present. The actual synovial joint region is the main portion of the SIJ and is located posteriorly; thus, the synovialis of the SIJ can be detected via US. Abnormal signals within and surrounding the SIJ may indicate synovitis of the SIJ (within the SIJ) or enthesitis of the ISL or the PSL (surrounding the SIJ) (Fig. 4B–D). Thus, US-CT fusion imaging was useful for locating the sites of abnormal vascularization in the SIJs of AS patients with active sacroiliitis and was therefore valuable for detecting intraligamentous inflammation and intracapsular inflammation at different levels of SIJ.

One limitation of this study was that we were not able to compare US-CT and US-MRI fusion images of the SIJ because of several technical limitations of US-MRI fusion and the low rate of patient consent to perform contrast-enhanced MRI, which is sufficiently sensitive to depict active inflammatory lesions associated with AS^18^,^19^. This comparison should be performed in our future studies. Moreover, we were not able to evaluate level 4 or 5 of the SIJ because the sizes of the fourth and fifth posterior sacral foramina were too small to be distinguished via US and because only a limited portion of the SIJ appeared in cross-sections of levels 4 and 5. Morphologic variation of the SIJ was not considered as a potential factor impacting the success rate of the image fusion process.

In conclusion, US-CT fusion imaging can be used to examine the SIJs of AS patients and to locate abnormal blood flow signals in the SIJs of AS patients with active sacroiliitis as visualized by CDUS. Abnormal blood flow signals at different levels indicate the occurrence of inflammation in different tissues of the posterior SIJ. US-CT imaging may provide a new visual perspective for imaging performance on AS patients and may be helpful in the diagnosis of AS patients, particularly at the early stages of AS.

## Methods

### Subjects

Between December 2013 and September 2014, 84 inpatients (168 SIJs), including 62 men and 22 women ranging in age from 17 to 42 years (26.1 ± 6.2), were recruited from our rheumatology department. All cases were diagnosed as AS (with bilateral sacroiliac abnormalities) by two experienced rheumatologists. Additionally, 30 controls (60 SIJs), including 16 men and 14 women ranging in age from 19 to 30 years (24.8 ± 3.2), were recruited from the hospital staff. The control subjects had no history of LBP. This study was approved by the ethics committee of Shanghai Jiaotong University Affiliated Sixth People’s Hospital, and the procedures were performed in accordance with approved guidelines. Written informed consent for the image fusion study was obtained from all patients and controls.

The 168 SIJs of the 84 inpatients were divided into the following 4 lesion classes by applying the modified New York criteria to the abnormalities that were visible on the CT images^20^: Class 1, suspicious changes but normal joint space; Class 2, definite early changes, including focal erosions, sclerosis and blurring of the joint margins, and equivocal joint space narrowing; Class 3, severe changes compared with Class 2 and definite joint space narrowing, possibly accompanied by small bony bridges or partial ankylosis; and Class 4, pronounced joint fusion and bony ankylosis.

### Cadavers

Three formalin-soaked cadaveric pelvises were used to investigate the soft tissues in the posterior portion of the SIJ and to perform the interventional test. All cadaveric pelvises were provided by the Department of Anatomy of Shanghai Jiao Tong University School of Medicine.

### Techniques

CT imaging was performed at our radiology department 3 days (at most) before image fusion (the participants received no medication during this period). The main imaging parameters of the CT scanner (GE LightSpeed VCT, Milwaukee, WI, USA) were as follows: slice thickness and interval, 0.625 mm; matrix, 512 × 512; detector collimation, 64×0.625 mm; tube voltage, 120 kV; and tube current–time product, 400 mA·s. CT data were imported to the US scanner for image fusion by CD-ROM.

Fusion imaging was performed using a virtual navigation system (Virtual Navigator 4.2, Esaote) incorporated into the US scanner (My-Lab90; Esaote, Genova, Italy). This system is a tracking system consisting of an electromagnetic transmitter and a receiver attached to the US probe (curved-array transducer, CA431, 1–8-MHz); the tracking system provides the orientation and the position of the probe in relation to the transmitter. The transmitter was placed at an appropriate distance (5 to 70 cm) from the receiver.

Image registration is the key process for fusing SIJ images. To initiate the registration procedure, the patients were placed in the prone position, and the probe was placed at the back of the sacral bone to search for the cross-sectional slice of the SIJ that fit over the corresponding CT image. Tuning mode, which uses internal marks that are visible in either modality, ensured that the US and CT images were optimally superimposed. In this study, we used 3 internal marks per plane to ensure precise co-registration. The internal marks were selected as needed from the following bony contours: the median sacral crest, the intermediate sacral crest, the lateral sacral crest, and the bony dorsal border of the iliac bone (Fig. 1A). The final fusion image was obtained when the 3 internal marks were accurately matched between the US and CT images. When the final image was formed, the software automatically calculated the registration accuracy and displayed one of following three virtual lights: green, indicating a registration error <0.5 cm; yellow, indicating a registration error >0.5 cm and <1 cm; and red, indicating a registration error >1 cm. We considered image fusion successful when the virtual light was green; a yellow or red virtual light indicated unsuccessful fusion.

Image fusion was performed on cross-sections of the first, second, and third posterior sacral foramina (defined as levels 1, 2, and 3, respectively). Before initiating image fusion, the skinfold thicknesses of the sacral region were measured at each level via US (linear array transducer, LA523, 5-12-MHz). To reduce measurement error, a water pad was placed between the skin and the probe.

After successful image fusion, CDUS was performed to detect abnormal blood flow signals and to estimate their positions. The located abnormal signals were grouped into 3 regions according to the 3 levels. The interior of the SIJ and the area surrounding the SIJ were used to describe the location of these signals cross-sectionally.

One of the 3 cadaveric SIJs was opened to obtain a complete view of the tissues within and surrounding the joint. Another cadaveric SIJ was sliced at cross-sections of the first, second, and third posterior sacral foramina (levels 1, 2, and 3, respectively) to investigate the tissue distribution at the different levels. The interventional test was performed on the third cadaveric SIJ; steel balls (1.5 mm) were implanted at different depths of the posterior portion of the SIJ at all 3 levels using a lumbar puncture needle (14G) under US guidance, and the joint was opened to confirm the positions of the balls. Finally, sections of soft tissue surrounding the steel balls were histologically examined.

### Statistical analysis

Inter-group and between-group differences were analyzed using the chi-square test or the t-test (SPSS 16.0). P values <0.05 were considered statistically significant. Abnormal blood flow signals detected by CDUS using the image fusion model were located, and differences between the groups were assessed using the chi-squared test (SPSS 16.0). P values <0.05 were considered statistically significant.

## Additional Information

**How to cite this article**: Hu, Z. *et al.* Feasibility of US-CT image fusion to identify the sources of abnormal vascularization in posterior sacroiliac joints of ankylosing spondylitis patients. *Sci. Rep.* **5**, 18356; doi: 10.1038/srep18356 (2015).

## Figures and Tables

**Table 1:** The baseline characteristics of the study patients and controls.

	AS patients (n = 84)	Controls (n = 30)
Age (years)	26.1 ± 6.2	24.8 ± 3.2
M/F	62/22	16/14
CRP(mg/dl)	5.5 ± 4.1	2.1 ± 1.2
ESR(mm/h)	31.5 ± 22.6	8.6 ± 5.9
BASDAI	4.4 ± 2.2	–

**Table 2:** Comparison of the skinfold thickness of the sacral region between successful and failed cases at each level in the AS and control groups.

		Successful cases	Failed cases	χ^*2*^	P
AS Group	Level 1	8.76 ± 2.48	4.50 ± 0.43	3.828	<0.001
	Level 2	9.05 ± 2.24	4.92 ± 0.62	8.369	<0.001
	Level 3	9.28 ± 2.11	5.21 ± 0.79	11.034	<0.001
Control	Level 1	9.48 ± 2.23	–	–	–
	Level 2	9.73 ± 2.00	6.23 ± 2.64	3.139*(t)*	0.004
	Level 3	9.80 ± 1.90	5.28 ± 0.86	4.649*(t)*	<0.001

**Table 3:** Comparison of image fusion success rates among the 4 classes of each level in the AS group.

		Failed cases	Successful cases	Success rates (%)	χ2	P
Level 1	Class				5.551	0.136*
	1	3	89	96.7		
	2	0	50	100.0		
	3	2	18	90.0		
	4	0	6	100.0		
Level 2	Class				1.391	0.708
	1	9	83	90.2		
	2	8	42	84.0		
	3	3	17	85.0		
	4	1	5	83.0		
Level 3	Class				3.574	0.311*
	1	14	78	84.8		
	2	14	36	72.0		
	3	5	15	75.0		
	4	1	5	83.3		

**Table 4:** Positions of the abnormal blood signals in the fused images of the SIJs.

	within	surround	total
Region 1	42	86	128
Region 2	41	64	105
Region 3	34	27	61

SIJ: Sacroiliac joint.
